# Optical coherence tomography and optical coherence tomography angiography findings in optic nerve hypoplasia and their relationships with visual acuity

**DOI:** 10.1038/s41598-024-57118-7

**Published:** 2024-03-26

**Authors:** Min Chae Kang, Kyung-Ah Park, Sei Yeul Oh

**Affiliations:** Department of Ophthalmology, Samsung Medical Center, Sungkyunkwan University School of Medicine, 81 Irwon-ro, Gangnam-gu, Seoul 06351 South Korea

**Keywords:** Deep retinal capillary plexus, Intraretinal microvasculature, Optical coherence tomography, Optical coherence tomography angiography, Optic nerve hypoplasia, Radial peripapillary capillary segment, Retinal nerve fiber layer, Superficial retinal capillary plexus, Optic nerve diseases, Vision disorders, Hereditary eye disease

## Abstract

This study aimed to quantitatively assess the thickness of the peripapillary retinal nerve fiber layer (pRNFL) thickness, as well as the microvascular alterations in the macula and peripapillary regions, in optic nerve hypoplasia (ONH) patients compared to normal controls. This was achieved through the utilization of spectral-domain optical coherence tomography (SD-OCT) and optical coherence tomography angiography (OCTA), with a specific focus on elucidating the association between these structural alterations and visual acuity. We included a total of 17 eyes of 12 ONH patients, and 34 eyes of age-matched 34 healthy controls. The pRNFL thickness was quantified using SD-OCT, while OCTA facilitated the visualization and measurement of the microvascular structure images of the superficial retinal capillary plexus (SRCP), deep retinal capillary plexus (DRCP), and radial peripapillary capillary (RPC) segment in the macula and peripapillary area. pRNFL thickness was measured for eight sectors (superior, temporal, inferior, nasal, superotemporal, superonasal, inferotemporal, and inferonasal). SRCP, DRCP, and RPC were measured for four sectors (superior, temporal, inferior, and nasal). Age, gender, and spherical equivalent refractive errors were statistically adjusted for the analysis. Associations of structural parameters with visual acuity in ONH patients were analyzed using Spearman correlation analysis. pRNFL thickness was significantly thinner in ONH patients than in controls for all sectors. Vessel densities of temporal and nasal sectors in DRCP were significantly higher in ONH patients, but vessel densities of the inferior sector in RPC were significantly lower than those in controls. For all sectors, pRNFL thickness was strongly associated with visual acuity in ONH patients. ONH patients showed significant pRNFL thinning and microvascular alterations compared to controls, and pRNFL thickness was strongly associated with visual function. OCT and OCTA are useful tools for evaluating optic disc hypoplasia and its functional status.

## Introduction

Optic nerve hypoplasia (ONH) is a congenital anomaly of multifactorial origin characterized by a decreased number of optic axons and an underdeveloped optic nerve with a visual function ranging from mildly subnormal vision to blindness^1^. ONH represents a significant etiology of pediatric blindness^2^. Its prevalence has been estimated to vary from 10.9/100,000 to 17.3/100,000^3,4^. ONH can occur unilaterally or bilaterally and may be an isolated finding or accompanied by other central nervous system abnormalities, most notably septo-optic-dysplasia (SOD)^5^.

ONH is diagnosed clinically with ophthalmoscopic confirmation of a small optic disc. Several funduscopic findings of ONH patients including tortuous retinal vessels, a “double ring sign” which consists of an outer ring (a normal junction between the sclera and the lamina cribrosa, and an inner ring (an abnormal extension of the retina and pigment epithelium over the outer portion of the lamina cribrosa) have been reported^6^. However, these funduscopic findings can be confusing since abnormally straight retinal vessels with reduced branching in ONH patients have been documented^7^. In addition, a double ring sign does not define ONH, as a similar appearance might be present in other conditions such as myopia^6^. Furthermore, ophthalmoscopic evaluation in the pediatric population with ONH can be challenging, making diagnostic confirmation difficult^6^.

Spectral-domain optical coherence tomography (SD-OCT) is a noninvasive imaging tool for acquiring detailed in vivo images of the optic nerve head, comparable to histologic samples^8^. To date, few studies have investigated structural alterations of ONH using OCT. Pilat et al. have reported thinning of retinal layers in 16 ONH patients using SD-OCT^5^. Moon et al. have described thinning of peripapillary retinal nerve fiber layer (pRNFL) and inner retinal layer using SD-OCT in one ONH patient compared with opposite healthy eye^9^. The recent advancements in optical coherence tomography angiography (OCTA) have been instrumental in our study, enabling detailed visualization of three-dimensional vascular structures of retinal layers and peripapillary regions by auto-segmentation, and provided us with quantitative data on vessel density but also guaranteed consistent repeatability of these measurements^10^. To our best knowledge, there are few reports of OCTA findings in congenital disc anomalies^11,12^. However, such studies of ONH patients have not been reported yet.

Thus, the aim of this study was to evaluate pRNFL thickness and macular and peripapillary microvascular alterations in ONH patients, comparing these metrics to healthy controls using SD-OCT and OCTA. Characteristics between intraretinal structural alterations in ONH were also determined. Furthermore, we investigated associations of these structural changes in SD-OCT and OCTA with visual functions in ONH patients.

## Methods

This retrospective cross-sectional study was approved by the Institutional Review Board (IRB) of Samsung Medical Center (Seoul, Republic of Korea, IRB No. 2022-10-014). It was conducted in accordance with the Declaration of Helsinki. This study included patients with ONH who visited the Department of Neuro-ophthalmology at the Samsung Medical Center from January 2015 to October 2022 and underwent OCT and OCTA between September 2015 and October 2022. Informed consent was waived by the ethics committee of the Institutional Review Board (IRB) of Samsung Medical Center. Patient information that could exactly identify a patient was removed prior to the data analysis.

The diagnosis of ONH was made based on clinical assessment with fundus photography. Funduscopic characteristics of ONH such as small optic disc, peripapillary “double-ring sign”, and tortuosity of retinal vasculature were observed in ONH patients. In addition, the manual Zeki method with fundus photography was used to diagnose ONH^13^. The average disc diameter (DD) was calculated by adding vertical and horizontal disc diameters and dividing by two. The distance from the temporal margin of the optic disc to the fovea was measured and half of the average DD was added to gain the optic disc to macula distance (D-M). The D-M/DD ratio was calculated and a ratio of three or more suggested ONH diagnosis in combination with subnormal visual acuity and/or visual field defects.

Thirty-four disease-free age-matched subjects were recruited as healthy volunteers who had undergone routine eye examinations. None of these controls had a history of ocular or neurologic disease. Healthy controls were required to have normal visual acuity, normal intraocular pressure ≤ 21 mmHg, and normal optic discs. Patients or healthy controls with other ophthalmic diseases (glaucoma, a refractive error greater than 6.0 diopters of spherical equivalent or 3.0 diopters of astigmatism in either eye, retinal disease, or any optic neuropathy other than ONH), previous retinal surgery or laser treatment, and those who were diagnosed with systemic/inflammatory diseases that could affect the thickness or vessel densities of the intra-retinal layer were excluded. In healthy controls, only right eye data were analyzed except when the data quality of the right eye was inappropriate while the data quality of the left eye was appropriate for the analysis of retinal layer thickness. Mydriatic eye drops (0.5% tropicamide and 0.5% phenylephrine hydrochloride) were used to dilate the pupil sufficiently to obtain SD-OCT and OCTA images successfully.

**Figure 1:**
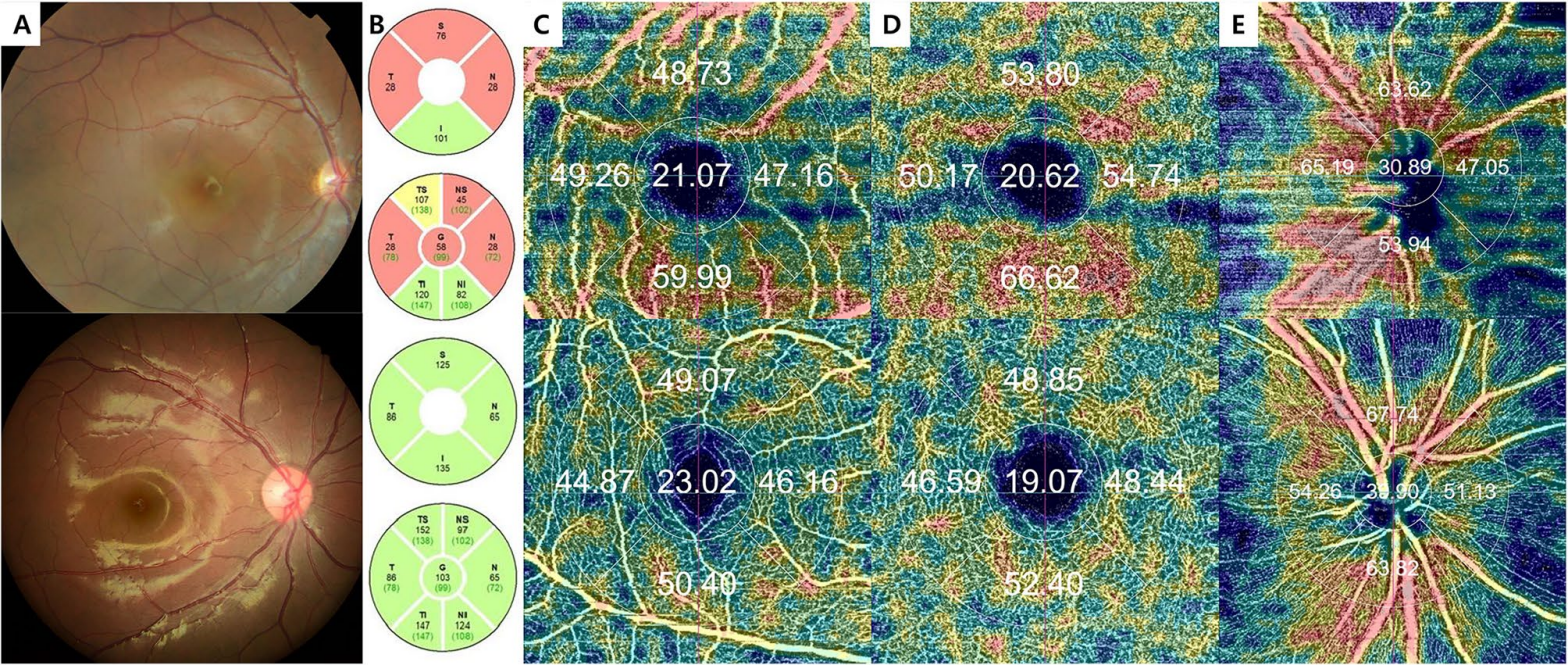
Representative images of OCT and OCTA of optic nerve hypoplasia patient (top row) and normal control (bottom row). (**A**) Fundus photography; (**B**) pRNFL thickness (µm) in OCT; (**C**) SRCP vessel densities (% area) in OCTA; (**D**) DRCP vessel densities (% area) in OCTA; (**E**) RPC vessel densities (% area) in OCTA. *OCT* optical coherence tomography; *OCTA* optical coherence tomography angiography; *pRNFL* peripapillary retinal nerve fiber layer; *SRCP* superficial retinal capillary plexus; *DRCP* deep retinal capillary plexus; *RPC* radial peripapillary capillary.

All subjects were scanned using fundus color photography and spectral domain OCT (SD-OCT, Spectralis, Heidelberg Engineering, Heidelberg, Germany) that provided 40,000 A-scans per second with 7 μm optical and 3.5 μm digital axial resolution. An automatic, real-time mode with active eye-tracking was used to automate eye alignment (TruTrack Active Eye Tracking; Heidelberg Engineering). We obtained OCT pRNFL thickness measurements from a 3.4-mm circular scans centered on the optic disc of each patient (Fig. 1). Microvasculatures of retina and peripapillary were analyzed using a Topcon OCT instrument (DRI OCT Triton Plus) for all of the subjects. The detailed protocol of measurements was described in a previous study^14^. The triton swept-source OCT was employed, operating at a wavelength of 1050nm and a scan speed of 100,000 A-scans/second. A set of three B-scans, which incorporating 500 A-scans each, were repeatedly recorded at 500 consistent locations for each field scan in a sequential manner to ensure the consistency of vessel density measurements. The time interval between consecutive B-scans was approximately 5 ms, this duration was factored in the mirror scan duty cycle. The Triton OCT system includes an eye-tracker to maintain synchronization with the patient’s fixation movements. It possesses the capability to recognize blinking and adjust the scan position to match, thus minimizing motion artifacts during OCTA image creation. Two imaging sessions were undertaken for each patient, consisting of a peripapillary scan (4.5 mm × 4.5 mm) centralized on the optic disc and a 3.0 mm × 3.0 mm perifoveal scan centralized on the macula. Superficial and deep retinal capillary plexuses were automatically segregated using the built-in layer segmentation feature on the OCT instrument software (IMAGEnet 6 V.1.14.8538). The system conducted automated segmentation, offering various predetermined reference boundaries for en-face projection and providing a final depth scale 2.6 µm/voxel in the output. The evaluations conducted with swept-source OCTA included vessel density, foveal avascular zone area, vascular abnormalities such as dilated capillary endings, and the quantity of choriocapillaris flow voids. The superficial retinal capillary plexus (SRCP) stretched from 3 µm below the internal limiting membrane (ILM) to 15 µm below the inner plexiform layer (IPL), while the deep retinal capillary plexus (DRCP) extended from 15 to 70 µm beneath the IPL, conforming to the established methodology of Park et al.^15^ The radial peripapillary capillary (RPC) segment extended from the ILM to the posterior boundary of the RNFL. Vessel density was established as the proportion of area occupied by vessels in a specified region. The software automatically fitted a 3 mm macular circle at the foveal center and generated vessel density for each layer with high repeatability and reproducibility^10,16^. Additionally, a 3 mm circle was manually adjusted at the disc center. Pericentral vessel density was calculated as the average of four quadrants within the macular circle, excluding the central circular zone. Five areas (center, nasal, temporal, superior, and inferior), dividing the center of the macula and disc, are depicted. The vessel density of each area was expressed as a percentage. Eyes with an image quality below 40, or exhibiting a partial decrease in image intensity, were disqualified. Images exhibiting significant eye movements during capture, indicated by motion artifacts involving more than three lines, discontinuities in blood vessels in OCTA images, or any inaccuracies in the location of the optic disc margin, were also excluded. Adjustments to the margin were performed manually, and verified by two trained graders (M.C.K and K.A.P) (Fig. 1).

Data are presented as mean ± standard deviation (SD). The chi-square test, Fisher’s exact test, and Wilcoxon rank sum test were used to compare gender, laterality, visual acuity, age, and spherical equivalent refractive errors (SER) between ONH patients and healthy controls. To compare measurements between ONH patients and controls, the generalized estimating equation was used with adjustments for age and SER. Benjamini–Hochberg correction was used for comparing multiple outcomes of SD-OCT and OCTA measurements. Spearman correlation analysis was used to evaluate associations of SD-OCT and OCTA parameters with visual acuity in ONH patients. A *p*-value of less than 0.05 was considered statistically significant. All statistical analyses were performed using R Statistical Software (version 3.6.3; Foundation for Statistical Computing, Vienna, Austria).

### Ethical approval

This retrospective cross-sectional study was approved by the Institutional Review Board (IRB) of Samsung Medical Center (Seoul, Republic of Korea, IRB No. 2022-10-014) and carried out in accordance with the principles of Declaration of Helsinki.

## Results

This study enrolled 17 eyes of 12 subjects with ONH and 34 eyes of 34 healthy controls. The mean logMAR visual acuity at the latest visit was significantly worse for patients than for healthy controls (0.47 ± 0.50 vs. 0.01 ± 0.05, *p* < 0.0001). There was no significant difference in gender, age at the time of OCT or OCT-A, or SER between the two groups (Table 1).

**Table 1:** Demographics of patients with optic nerve hypoplasia and controls.

	All subjects	ONH patients	Controls	*p* value (ONH versus Controls)
No. of eyes (No. of subjects)	51 (46)	17 (12)	34 (34)	–
Female (%)	22 (43.1%)	9 (52.9%)	13 (38.2%)	0.4841^a^
Laterality				
OD (%)	42 (82.4%)	11 (64.7%)	31 (91.2%)	**0.0455**^b^
OS (%)	9 (17.6%)	6 (35.3%)	3 (8.8%)	
Visual acuity (logMAR) (mean ± SD)	0.15 ± 0.34	0.47 ± 0.50	0.01 ± 0.05	** < 0.0001**^c^
Age at the time of OCT, years (mean ± SD)	23.97 ± 16.42	24.74 ± 17.07	23.60 ± 16.35	0.9917^c^
Age at the time of OCTA, years (mean ± SD)	24.05 ± 16.09	24.78 ± 16.30	23.68 ± 16.23	0.9442^c^
SER (SD)	−3.42 (2.79)	−4.35 (3.62)	−2.95 (2.19)	0.2570^c^

*ONH* optic nerve hypoplasia; *No.* number; *OD* oculus dexter (right eye); *OS* oculus sinister (left eye); *SD* standard deviation; *OCT* optical coherence tomography; *OCTA* optical coherence tomography angiography; *SER* spherical equivalent refractive errors.

^a^Chi-square test; ^b^Fisher’s exact test; ^c^Wilcoxon rank sum test.

Bold value indicates *p* < 0.05.

### Comparison of pRNFL thickness between ONH patients and the control group

pRNFL thickness measured by SD-OCT was significantly thinner in ONH patients than in controls after adjusting for age and SER (estimates, *p*-value: average, −39.173, *p* < 0.0001; superior, −39.173, *p* < 0.0001; temporal, −49.905, *p* = 0.0106; inferior, −37.333, *p* = 0.0017; nasal, −40.237, *p* < 0.0001; supero-temporal, −51.504, *p* = 0.0001; supero-nasal, −53.203, *p* < 0.0001; infero-temporal, −31.578, *p* = 0.0477; infero-nasal, −36.238, *p* = 0.0007) (Table 2).

**Table 2:** Comparisons of spectral domain-optical coherence tomography measures in eyes of optic nerve hypoplasia patients and controls.

	ONH patients (*n* = 17)	Controls (*n* = 34)	Univariable analysis			Multivariable analysis^b^		
			Estimate	95% CI	*p* value^a^	Estimate	95% CI	*p* value^a^
pRNFL thickness (μm)								
Average	65.19 ± 30.77	103.31 ± 7.99	−38.121	−55.853, −20.390	**0.0001**	−39.173	−56.906, −21.441	** < 0.0001**
Superior	75.50 ± 33.21	125.29 ± 14.07	−49.794	−69.802, −29.787	** < 0.0001**	−49.905	−71.629, −28.180	** < 0.0001**
Temporal	63.63 ± 39.08	90.88 ± 15.80	−27.257	−49.812, −4.703	**0.0201**	−29.269	−51.352, −7.185	**0.0106**
Inferior	88.69 ± 41.84	124.94 ± 17.87	−36.254	−60.236, −12.272	**0.0039**	−37.333	−60.121, −14.545	**0.0017**
Nasal	33.19 ± 21.79	72.41 ± 15.34	−39.224	−52.810, −25.639	** < 0.0001**	−40.237	−52.136, −28.338	** < 0.0001**
ST	96.00 ± 40.25	145.26 ± 24.20	−49.265	−73.442, −25.087	**0.0001**	−51.504	−76.649, −23.360	**0.0001**
SN	54.06 ± 28.44	107.41 ± 16.23	−53.349	−71.352, −35.347	** < 0.0001**	−53.203	−72.437, −33.969	** < 0.0001**
IT	114.25 ± 50.27	144.44 ± 29.42	−30.191	−60.429, 0.047	0.0503	−31.578	−62.839, −0.317	**0.0477**
IN	63.00 ± 36.36	98.76 ± 26.79	−35.765	−56.916, −14.613	**0.0014**	−36.238	−56.607, −15.869	**0.0007**

*ONH* optic nerve hypoplasia; *n* numbers; *pRNFL* peripapillary retinal nerve fiber layer; *ST* superotemporal; *SN* superonasal; *IT* inferotemporal; *IN* inferonasal; *CI* confidence interval.

Values are shown as mean ± standard deviation.

Estimate and 95% CI were calculated by Generalized Estimating Equation (GEE).

^a^*p* value was calculated by Benjamini-Hochberg correction for multiple outcomes.

^b^Multivariable analysis with adjustment for age and spherical equivalent refractive errors.

Bold value indicates *p* < 0.05.

### Comparison of vessel densities between ONH patients and the control group

Vessel densities of DRCP were significantly higher in ONH patients than in controls (average, 2.693, *p* = 0.0120; temporal, 3.770, *p* = 0.0086; nasal, 4.934, *p* = 0.0043). In the temporal sector of SRCP, vessel densities were higher in patients than in controls, although the significance was only marginal (temporal, 2.874, *p* = 0.0581). In contrast, vessel density in the inferior sector of RPC segments was significantly lower in ONH patients than in controls (inferior, -10.848, *p* = 0.0001) (Table 3).

**Table 3:** Comparisons of optical coherence tomography angiography measures in eyes of optic nerve hypoplasia patients and controls.

	ONH patients (*n* = 17)	Controls (*n* = 34)	Univariable analysis			Multivariable analysis^b^		
			Estimate	95% CI	*p* value^a^	Estimate	95% CI	*p* value^a^
SRCP, % area								
Average	50.03 ± 4.23	48.64 ± 1.86	1.386	−0.751, 3.523	0.3055	1.278	−0.753, 3.310	0.2175
Superior	49.58 ± 5.75	49.52 ± 2.20	0.052	−2.832, 2.936	0.9716	0.455	−2.172, 3.081	0.7342
Temporal	51.46 ± 5.83	48.30 ± 2.77	3.163	0.158, 6.168	0.1066	2.874	0.465, 5.283	0.0581
Inferior	48.18 ± 5.65	49.07 ± 3.68	−0.893	−3.936, 2.150	0.6783	−0.982	−4.311, 2.347	0.6759
Nasal	50.90 ± 6.61	47.67 ± 2.67	3.222	−0.046, 6.490	0.1066	2.766	0.006, 5.527	0.0990
DRCP, % area								
Average	53.55 ± 4.12	50.80 ± 2.78	2.749	0.361, 5.136	**0.0481**	2.693	0.704, 4.682	**0.0120**
Superior	53.20 ± 4.52	52.69 ± 4.80	0.518	−2.057, 3.094	0.6931	0.538	−2.155, 3.231	0.6955
Temporal	53.25 ± 6.68	49.48 ± 4.89	3.774	0.151, 7.398	0.0618	3.770	1.184, 6.357	**0.0086**
Inferior	53.57 ± 5.25	51.96 ± 5.14	1.601	−1.810, 5.011	0.4291	1.529	−2.022, 5.081	0.4784
Nasal	54.17 ± 6.58	49.07 ± 3.46	5.102	1.467, 8.736	**0.0178**	4.934	1.898, 7.970	**0.0043**
RPC, % area								
Average	56.20 ± 8.02	60.38 ± 2.89	−4.178	−8.159, −0.197	0.0793	−4.576	−8.819, −0.332	0.0691
Superior	64.26 ± 11.31	69.40 ± 4.88	−5.147	−10.584, 0.291	0.0953	−5.697	−11.752, 0.359	0.0978
Temporal	52.40 ± 13.73	51.34 ± 5.27	1.064	−6.572, 8.699	0.7849	1.288	−5.674, 8.250	0.7169
Inferior	61.14 ± 13.13	70.17 ± 5.36	−9.033	−15.549, −2.517	**0.0198**	−10.848	−15.775, −5.921	**0.0001**
Nasal	47.01 ± 13.10	50.60 ± 5.18	−3.597	−11.015, 3.821	0.4103	−3.045	−11.810, 5.719	0.5950

*ONH* optic nerve hypoplasia; *n* numbers; *SRCP* superficial retinal capillary plexus; *DRCP* deep retinal capillary plexus; *RPC* radial peripapillary capillary; *CI* confidence interval.

Values are shown as mean ± standard deviation.

Estimate and 95% CI were calculated by Generalized Estimating Equation (GEE).

^**a**^*P* value was calculated by Benjamini–Hochberg correction for multiple outcomes.

^b^Multivariable analysis with adjustment for age and spherical equivalent refractive errors.

Bold value indicates *p* < 0.05.

### Associations of pRNFL thickness, vessel densities, with final visual acuity in ONH patients

**Figure 2:**
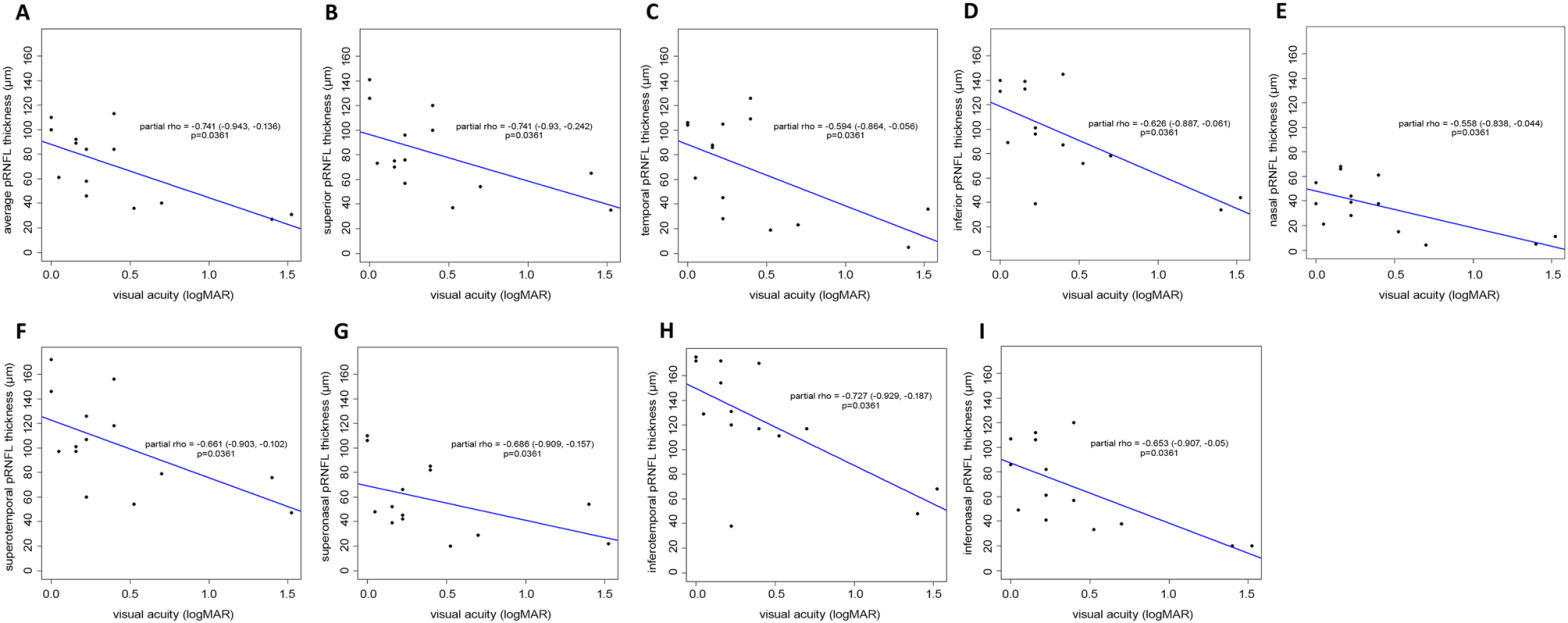
Scatter plots showing significant associations of spectral domain-optical coherence tomography parameters of pRNFL thickness with visual acuity in optic nerve hypoplasia patients. (**A**) Significant association between thicker average pRNFL and better visual acuity (r = −0.741, *p* = 0.0361); (**B**) Significant association between thicker superior pRNFL and better visual acuity (r = −0.741, *p* = 0.0361); (**C**) Significant association between thicker temporal pRNFL and better visual acuity (r = −0.594, *p* = 0.0361); (**D**) Significant association between thicker inferior pRNFL and better visual acuity (r = −0.626, *p* = 0.0361); (**E**) Significant association between thicker nasal pRNFL and better visual acuity (r = −0.558, *p* = 0.0361); (**F**) Significant association between thicker superotemporal pRNFL and better visual acuity (r = −0.661, *p* = 0.0361); (**G**) Significant association between thicker superonasal pRNFL and better visual acuity (r = −0.686, *p* = 0.0361); (**H**) Significant association between thicker inferotemporal pRNFL and better visual acuity (r = −0.727, *p* = 0.0361); (**I**) Significant association between thicker inferonasal pRNFL and better visual acuity (r = −0.653, *p* = 0.0361). pRNFL, peripaillary retinal nerve fiber layer.

For all sectors, thicker pRNFL thickness was significantly associated with better visual acuity of ONH patients with age and SE adjustments by Spearman correlation analysis (average, r = -0.741, *p* = 0.0361; superior, r = −0.741, *p* = 0.0361; temporal, r = −0.594, *p* = 0.0361; inferior, r = −0.626, *p* = 0.0361; nasal, r = −0.558, *p* = 0.0361; supero-temporal, r = −0.661, *p* = 0.0361; supero-nasal, r = −0.686, *p* = 0.0361; infero-temporal, r = −0.727, *p* = 0.0361; infero-nasal, r = −0.653, *p* = 0.0361). Among sectors, pRNFL thicknesses of superior and inferotemporal sectors were strongly associated with visual acuity (Table 4, Fig. 2). Associations between OCTA measurements and visual acuity were also analyzed. There was no significant relationship between vessel densities measured with OCTA and visual acuity by Spearman correlation analysis (Table 4).

**Table 4:** Associations of spectral domain-optical coherence tomography and optical coherence tomography angiography parameters with Final visual acuity in optic nerve hypoplasia patients.

	Correlation			Partial correlation^c^		
	r	95% CI^a^	*p* value^b^	r	95% CI^a^	*p* value^b^
pRNFL thickness (μm)						
Average	−0.720	−0.905, −0.306	**0.0167**	−0.741	−0.943, −0.136	**0.0361**
Superior	−0.649	−0.877, −0.181	**0.0217**	−0.741	−0.930, −0.242	**0.0361**
Temporal	−0.498	−0.814, 0.044,	0.0697	−0.594	−0.864, −0.056	**0.0361**
Inferior	−0.649	−0.877, −0.181,	**0.0217**	−0.626	−0.887, −0.061	**0.0361**
Nasal	−0.616	−0.864, −0.128	**0.0283**	−0.558	−0.838, −0.044	**0.0361**
ST	−0.604	−0.859, −0.108	**0.0284**	−0.661	−0.903, −0.102	**0.0361**
SN	−0.507	−0.818, −0.032	0.0697	−0.686	−0.909, −0.157	**0.0361**
IT	−0.749	−0.916, −0.363	**0.0167**	−0.727	−0.929, −0.187	**0.0361**
IN	−0.681	−0.890, −0.235,	**0.0217**	−0.653	−0.907, −0.050	**0.0361**
SRCP, % area						
Average	−0.352	−0.744, 0.219	0.4328	−0.331	−0.722, 0.220	0.4700
Superior	−0.228	−0.677, 0.344	0.4328	−0.178	−0.699, 0.466	0.6066
Temporal	0.241	−0.332, 0.684	0.4328	0.332	−0.419, 0.814	0.4711
Inferior	−0.487	−0.809, 0.058	0.2315	−0.582	−0.895, 0.114	0.2829
Nasal	−0.255	−0.692, 0.319	0.4328	−0.310	−0.745, 0.311	0.4711
DRCP, % area						
Average	0.288	−0.286, 0.710	0.4772	0.352	−0.394, 0.818	0.5380
Superior	−0.190	−0.655, 0.378	0.5142	−0.134	−0.590, 0.387	0.6276
Temporal	0.197	−0.372, 0.659	0.5142	0.177	−0.496, 0.717	0.6276
Inferior	−0.341	−0.738, 0.231	0.4654	−0.616	−0.924, 0.177	0.3499
Nasal	0.419	−0.144, 0.777	0.4089	0.475	−0.477, 0.914	0.5380
RPC, % area						
Average	−0.464	−0.789, 0.064	0.3015	−0.552	−0.862, 0.058	0.2189
Superior	−0.216	−0.655, 0.333	0.6603	−0.263	−0.735, 0.380	0.5740
Temporal	−0.410	−0.762, 0.130	0.3015	−0.457	−0.762, 0.014	0.2189
Inferior	−0.144	−0.611, 0.398	0.7311	−0.034	−0.408, 0.350	0.8670
Nasal	−0.390	−0.752, 0.153	0.3015	−0.426	−0.823, 0.250	0.4193

*pRNFL* peripaillary retinal nerve fiber layer; *ST* superotemporal; *SN* superonasal; *IT* inferotemporal; *IN* inferonasal; *SRCP* superficial retinal capillary plexus; *DRCP* deep retinal capillary plexus; *RPC* radial peripapillary capillary; *CI* confidence interval.

^a^95% CI for correlation coefficients using z-transformation.

^b^Spearman correlation analysis.

^c^Adjustment for age and spherical equivalent refractive errors.

Bold value indicates *p* < 0.05.

## Discussion

This study quantitatively evaluated alterations in the retinal nerve fiber layer and microvasculatures in ONH patients compared with age-matched healthy controls. Associations between these structural changes with visual function were also analyzed. Our study showed significant pRNFL thinning of all sectors in ONH patients compared to controls, consistent with the results of previous studies. Several previous studies have reported OCT findings in ONH patients. Pilat et al. have reported significant pRNFL (nasally) and ganglion cell layer (GCL) (nasally and temporally) thinning using SD-OCT in 16 ONH patients^5^. pRNFL and GCL thinning supported the theory of ONH occurring because of the retrograde RNFL atrophy during fetal development, as similar changes were described in optic nerve atrophy^5^. This could explain that the coexistence of SOD might be one possible causal factor of ONH^17,18^. Other studies have also reported results of pRNFL thinning in ONH patients using SD-OCT^9,19,20^.

We compared vessel densities of the macula and peripapillary regions measured by OCTA in ONH patients and controls. In SRCP, extending from 3 µm below the ILM to 15 µm below the IPL in the macula, there was only a marginally significant increase in vessel density in the temporal sector between the two groups. In DRCP, extending from 15 to 70 µm below the IPL in the macula, vessel densities of the temporal and nasal sectors were significantly higher in ONH patients than in controls. So far, vessel densities of ONH measured by OCTA have not been reported. A previous study using OCT has found thinning of pRNFL, but increased central retinal thickness in ONH because of the changes in GCL, IPL, and outer plexiform layer (OPL)^5^. Increased central macular thickness reported in the previous study as well as increased microvascular densities in this study suggest arrested foveal development in ONH, which has similar structures of foveal hypoplasia^5^. During normal prenatal and early postnatal periods, centrifugal movement of inner retinal layers leads to the formation of the foveal pit^5^. At the same time, the thickening of IS and OS layers and widening of ONL occur^21^. Continuation of IPL, OPL, and ONL in the fovea in ONH suggests arrested foveal development^5^. These findings are consistent with a previous report that studied OCT findings of 29 eyes of 18 ONH patients compared with 21 eyes of 21 controls, in which eyes with ONH showed 17 subnormal fovea (a normal foveal depression that seemed shallow due to thinning of the GCC around the fovea), 7 foveal hypoplasia, and 5 atypical fovea abnormality^20^. In contrast, microvascular densities of the inferior sector of RPC of peripapillary regions of ONH patients were significantly lower than in healthy controls. Our study also showed thinner pRNFL in ONH patients in all sectors, consistent with previous studies^5,9,20,22^. Reduced vessel densities especially in the inferior sector of peripapillary regions in this study might be correlated with findings of a previous histopathologic study of ONH, which reported that optic nerves of ONH showed severe axonal depletion without degenerated profiles in an inferonasal sector, with only a small superotemporal sector having a near normal appearance^1^.

We also analyzed the relationships between OCT and OCTA findings with the latest visual acuity in patients with ONH. We found a significant positive association between pRNFL thickness for all sectors and the latest visual acuity in ONH patients. A few studies have suggested useful parameters related to visual function in ONH patients, such as pupil reactivity^23^, nystagmus^23^, ratio of horizontal disc diameter to disc-macula distance^23–26^, orbital optic nerve diameter from MRI scans^24^, OCT parameters^22^, visual-evoked potential (VEP) threshold and amplitude^23,25,26^, and electroretinogram (ERG) parameters^25^. Skriapa-Manta et al. have recently reported relationships between OCT parameters and visual acuity^22^. They analyzed data from 37 eyes with ONH and found that thinner mean pRNFL and mean GCC were correlated to poor visual acuity and greater visual field defects^22^. Our study results were consistent with their findings. However, we found no correlations between macular and peripapillary vessel densities in OCTA and visual acuity in patients with ONH. Further studies are needed to reveal the clinical implications of microvascular alterations in ONH.

Our study has several limitations. First, we analyzed a small number of ONH patients. Thus, a further large-scale study is needed. Second, the quantification of vessel density was conducted using OCTA, in which the angiographic signal was based on movement. OCTA measurements encompassed large blood vessels, which might have increased the susceptibility of quantification bias. Alterations in vessel density could result from a combination of major vessels. Third, the ONH patients in this study were homogenous in ethnicity. Thus, the direct application of these data to other races might need caution.

This study was the first to quantify microvascular changes in ONH patients compared to healthy controls. We found significantly higher vessel densities in sectors of DRCP (average, temporal, nasal) in ONH with lower vessel densities in the inferior sector of RPC in ONH patients than in controls. We also demonstrated thinner pRNFL in patients with ONH and their significant relationship with visual function. These results suggest that OCT and OCTA can be useful tools for evaluating ONH and its functional status. Further research is needed to clarify the clinical significance of microvascular alterations in ONH.

## Data Availability

All data are available upon request from the corresponding author.
